# Selections and Abstracts

**Published:** 1913-08

**Authors:** 


### SELECTIONS AND ABSTRACTS

### THE SANITARY CONTROL OF VENEREAL DISEASES IN NEW YORK.*

There can be no question that the control of the venereal diseases is a public health problem of the first magnitude. These diseases are infectious and communicable, the direct and indirect cause of a vast amount of preventable illness and many deaths, and constitute ea serious menace to the physical stamina of the community and the nation. The New York Board of Health many years ago laid down the principle that all infectious and preventable diseases and everything pertaining to their diagnosis, supervision and cure fell within the proper functions

*Monthly Bulletin of the Dept. of Health of the City of New York, June, 1913.

of the public health authorities. It might have been expected that a department which led in the administrative control of tuberculosis, diphtheria and other diseases, would long ago have undertaken the surveillance of venereal diseases, and only the perplexing social and moral problems involved have prevented such action. Finally, however, the Board of Health became convinced that the time had come to intervene. It was felt that police regulation had been a failure everywhere and would remain such and that the only possibility of effecting a reduction in the prevalence of venereal diseases lay in education and rational supervision by the public health authorities.

POLICE SURVEILANCE.

Reglementation or police regulation of prostitution holds the prevention of disease as the first object. Aside from the question whether the control of vice should not be considered even a more fundamental objective, it is proven by experience that reglementation with or without segregation is a failure from the sanitary point of view. As a system it succeeds best in controlling the least dangerous element, misses the clandestine prostitutes who include by far the larger number of diseased women, and accomplishes its little good at far too great a cost of discrimination against one sex, invasion of personal liberty, and official recognition and countenancing of the social evil. A hundred years trial in Paris under one of the most efficient police systems in the world, has failed to give results which justify this method of attacking the problem, and the experience of other European cities is much the same. Such success as has been attained seems to have depended upon the rigor with which the laws or police rules have been enforced, and upon the degree of support and approbation which the system has received from the community. In some foreign countries, especially in Hungary, and particularly in the city of Budapest, there seems no doubt that however detrimental such methods may have been to the morals of the cummunity, their rigid enforcement has resulted in a marked decrease in the number of venereal diseases. On the other hand, in certain American cities, such regulations have been little more than a farce, and have presumably been productive of more harm than good. In

English speaking countries, there has always been a very great aversion to the public discussion of venereal diseases, and a great reluctance to institute legislation or administrative systems seeking to diminish their prevalence by means of public regulation.

This fact may accompany or reflect a difference in the standard of morals recognized in these countries. The measures that have been advocated, and enforced with more or less strictness in non-English speaking countries, have generally disregarded any moral phase of the subject.

The opposition to the system of police surveillance, which originated in England in a long but successful campaign for the repeal of the Contagious Diseases Acts of 1866, has spread over Europe in the course of the past 50 years and has taken organic form in national and international conferences and associations for the abolition of reglementation in favor of broader, juster and more effective methods of dealing with the problem.

In Europe the Scandinavians were the first to approach this subject from the point of view of sanitary control by including venereal diseases among the infectious diseases required to be reported and supervised by the health authorities. Simliar action has been taken in Australia with respect to syphilis, and some American state governments are now moving in the same direction. Vermont has adopted this year a state law practically repeating the New York City regulations of 1912.

### In The United States.

Police regulation in this country under the old-fashioned European plan has been confined to a short experiment in St. Louis, official segregation in New Orleans, and a recent movement in San Francisco where a public clinic for examination and treatment was established in connection with regulations providing for segregation. In New York sharp opposition to the so-called "Clause 79" of the Page Law of 1910 which provided for examination of convicted prostitutes developed from a belief that this was an entering wedge of reglementalion. The matter was promptly taken into Court where this part of the law was finally declared unconstitutional.

### Beginning of Sanitary Control in New York.

The first official action looking toward a plan for the administrative control of venereal diseases by the Department of Health was taken when the Board of Health on April 4th, 1911, adopted the following resolutions:

> Resolved, That it is the sense of this Board that the protection of the public health requires the early adoption of measures for the sanitary control of venereal diseases by the Department of Health, and that the Medical Advisory Board, be, therefore, and hereby is requested to consider this subject, and submit recommendations for a comprehensive plan for the public control of said diseases, and
>
> Whereas, The establishment of a public hospital for the care of patients suffering from these diseases has been advised as the first step toward a system of sanitary control,
>
> Therefore be it further
>
> Resolved, That the Board of Health urge upon the Board of Estimate and Apportionment the need of making provision in the next annual Corporate Stock Budget for the erection, at an early date, of such hospital in the City of New York.

As the first step toward carrying out the policy thus announced, application was immediately made for an appropriation to construct a special pavilion at Riverside Hospital on North Brother Island. This request was favorably received and on July 17th, 1911, the Board of Estimate and Apportionment set aside the sum of $55,000 to be expended by the Department of Health for this purpose.

From the first it was definitely planned to postpone the requirement for reporting cases until provision for a special hospital should have been made. It was felt that as soon as any scheme of public surveillance went into effect the Department might be called upon to remove and detain certain flagrant and unruly cases as has been the experience in tuberculosis, and therefore it was necessary to preface any plan with the construction of a properly equipped hospital.

### Report of the General Medical Officer.

At a meeting of the Board on December 8th, 1911, Dr. Hermann M. Biggs, the General Medical Officer, submitted the following communication again calling attention to the necessity for adopting some measures for the sanitary supervision of venereal diseases:

"I desire to direct the attention of the Board to the fact that the time has arrived when, in my judgment, it becomes incumbent upon the Department of Health to adopt some measures for the sanitary surveillance of the venereal diseases. I fully realize how difficult and serious this problem is likely to prove, but all the attempts, which have been previously made in this country to deal with these diseases from the moral and social side, have utterly failed to effect the end in view. In the final analysis, so far as the public health authorities are concerned, this problem is a sanitary one and these diseases should be treated as other infectious and communicable diseases dangerous to the public health are treated. The moral and social aspects of the problem do not primarily concern the sanitary authorities. As to the important part which these diseases play in any great city in the production of sickness and death, there can be no question despite the fact that no accurate statistics are available.

"While the Board of Health has long felt keenly the importance of this problem, it has also realized that no effective attempts could be made with it unless a hospital for the isolation of such cases under its care was provided. The Board of Estimate and Apportionment has this year made an appropriation of $55,000 for the erection of a pavilion for eighty beds as an initial step in this direction. This appropriation was made in response to a request from the Board for an issue of $300,000 of corporate stock for the provision of suitable hospital accommodations. It is, therefore, now desirable that the Board of Health, in the early future, adopt such measures as seem expedient and desirable, looking to the administrative control of these diseases.

"Every system of administrative control of the infectious and communicable diseases must be based on the fullest information obtainable concerning the number and distribution of cases, and this information can be obtained only by notification and registration of such cases. Strenuous objections have frequently been made to the notification of the venereal diseases, and the real difficulties of the problem are undoubtedly very great, and many of the arguments advanced do not differ materially from those used and successfully refuted when the proposition for the notification of tuberculosis was first introduced. The additional argument in the case of venereal diseases, that notification places a stigma upon the moral character of the person involved and that it would lead to family dissensions, is largely disposed of by providing that the reports shall be treated as absolutely confidential.

In the venereal diseases, as in other communicable diseases, the danger to the public health is to a considerable extent dependent on the social position of the patient, and for the same reasons, namely, because the ignorant poor as well as the social outcasts, are unable or unwilling to secure proper medical care, and their persons and their suroundings are often extremely filthy and overcrowded. Moreover, as far as the venereal diseases are concerned, the persons who are most undesirable as hospital patients are frequently just the ones who constitute the gravest menace to others if left at large. Some hospital must, therefore, be provided where undesirable patients refused by or discharged from other institutions will be accepted. Such a hospital must be under the complete control of the public health authorities in order that, if need be, patients in such a condition that they constitute a serious menace to others if allowed at large, may be restrained in the hospital until this menace has disappeared or until it has at least been matrially lessened. Bearing in mind, then, this interrelation of social position and public health menace, we may logically begin the administrative control of the venereal diseases, in addition to an educational campaign, by work confined largely to the ignorant poor and to the social outcasts.

"When a person suffering from venereal dsease is being treated in a public institution, or is being cared for at the public expense, there is really no reason whatsoever for not at once reporting the case to the public health authorities. The latter then have the information to exercise such supervision over the patient, as may be possible and necessary, until the infection is no longer dangerously communicable. If the infected person is homeless, or if his or her occupation (e. g., prostitution) makes it dangerous to others for the person to be at large, the health authorities will order the patient's removal to the isolation hospital. If, however, this is not the case, and the person is able to assure the health authorities that the necessary treatment will be carried out, and the proper precautions will be taken to prevent infecting others, the health authorities may allow him or her to pursue his or her ordinary vocation provided he or she allows himself or herself to be kept under surveillance until the liability to infect others is no longer present.

"Cases under the care of private physicians may, in general, be assumed to be much less of a public health menace. This statement applies specially to patients continuing under the physician's care until the dangerously communicable lesions have healed.

"As in other infectious diseases, the public health authorities should provide every facility for promptly and accurately diagnosing the venereal infections. In the case of the gonorrhoeal infections, specially prepared smears should be examined for the presence of Gram-Negative intracellular diplococci. In the case of syphilis both microscopical diagnosis (for treponema) and serum reactions (Wassermann test) should be made available. As in other examinations in the diagnosis laboratory, no reports should be furnished unless the specimen be accompanied by the data necessary for the registration of the patient.

"Facilities should also be provided for supplying gonococcus vaccines to physicians, hospitals and dispensaries, and the salvarsan treatment of syphilis should be available for all suitable cases willing to take the treatment at the isolation hospital.

"Presenting to the medical profession of the city the great need of the administrative control of the venereal diseases, the public health authorities could ask for the hearty co-operation of all physicians and the authorities of all public institutions and request them to report to the Department of Health all cases of venereal diseases coming under their notice. The records should be strictly confidential and should under no condition be made public."

The Board of Health caused copies of the foregoing report together with tentative resolutions to be sent to each member of the Medical Advisory Board with a request for earnest consideration and the expression of individual opinion as to the wisdom of the contemplated action, preparatory to further discussion at a formal meeting of the Advisory Board.

### Regulations Adopted.

When finally approved by the Medical Advisory Board which includes a number of the most prominent physicians of New York the resolutions were duly adopted by the Board of Health on February 20, 1912, in the following form:

Whereas, The venereal diseases are infectious, communicable and preventable, and constitute a serious menace to the public health, thus properly coming under the charge of the public health authorities, and

Whereas, It is well established that no administrative control of such diseases is possible without a system of notification and registration, associated with provision for the municipal care of patients unable or unwilling to place themselves under proper medical care and to take the precautions necessary to prevent the infection of others, Be it therefore

Resolved, First, That on and after May 1, 1912, the superintendents or other officers of all public institutions such as hospitals, dispensaries, clinics, homes, asylums, charitable and correctional institutions, including all institutions which are supported in whole or in part by voluntary contributions, be required to report promptly the name, sex, age, nationality, race, marital state and address of every patient under observation suffering from syphilis, in every stage, chancroid, or gonorrhoeal infection of every kind (including gonorrhoeal arthritis), stating the name, character, stage and duration of the infection, the date and source of contraction of the infection, if obtainable, and,

Second, That all physicians be requested to furnish similar information concerning private patients under their care, excepting that the name and address of the patient need not be reported.

Third, That all information and all reports, in connection with persons suffering from these diseases, shall be regarded as absolutely confidential, and shall not be accessible by the public nor shall such records be deemed public records.

Fourth, That the Department of Health shall provide facilities for the free bacteriological examination of discharges for the diagnosis of gonorrhoeal infections, and also shall provide, without charge, vaccines for the treatment of such infections, and

Fifth, That the Department of Health shall undertake to make, without charge, the Wassermann and the Noguchi tests for the diagnosis of syphilis and examine specimens for spirochetes.

Sixth, That these diagnostic and therapeutic facilities be extended only when the data required for the registration of the case be furnished by the physician treating the patient, and

Seventh, That the Department provide and distribute circulars of information in relation to these diseases.

### First Statistical Results.

The reporting order above described went into effect May 1st, 1912.

Cases reported during the twelve months following were as follows, by diseases and months:

| 1912 | Syphilis | Gonorrhea | Chancroid | Total |
|---|---|---|---|---|
| May ............ | 100 | 101 | 16 | 217 |
| June ............ | 181 | 195 | 33 | 409 |
| July ............ | 365 | 533 | 42 | 940 |
| August .......... | 312 | 274 | 30 | 616 |
| September ....... | 379 | 439 | 60 | 878 |
| October . ........ | 351 | 419 | 45 | 815 |
| November ....... | 530 | 458 | 64 | 1,052 |
| December ....... | 267 (10) | 423 (5) | 44 (1) | 734 |
| 1913 | | | | |

| | | | | | | | |
|---|---|---|---|---|---|---|---|
| January | 960 | (24) | 509 | (8) | 32 | | 1,501 |
| February | 649 | (11) | 482 | | 52 | (1) | 1,183 |
| March | 762 | (15) | 825 | (7) | 58 | | 1,645 |
| April | 911 | (11) | 627 | (4) | 45 | | 1,583 |
| Totals | 5,767 | | 5,285 | | 521 | | 11,573 |

All figures in parenthesis denote out of town cases which are included in the totals for months given.

Owing to the pioneer character of the order and the large amount of opposition, known and unknown, to the requirement, it is of course not yet possible to derive an accurate idea of the extent of these diseases from statistics of cases officially reported. In order to provide the basis for a somewhat better estimate, the Department, in December, 1912, instituted a special inquiry by sending a circular to the 7,000 physicians of Greater New York, requesting a report merely of the number of cases observed in their private practice during the year 1912. The results of this special census were as follows:

| | |
|---|---|
| Number of physicians inquired of | 7,000 |
| Number of physicians responding | 2,217 |
| Number of cases reported as observed by physicians in 1912: | |
| Syphilis | 13,350 |
| Gonorrhea | 24,984 |
| Chancroid | 4,331 |
| | 42,665 |
| Number of institutions inquired of | 197 |
| Number of institutions responding | 10 |
| Number of cases reported as observed at institutions in 1912: | |
| Syphilis | 1,849 |
| Gonorrhea | 3,477 |
| Chancroid | 336 |
| | 5,662 |

### Prevalence of the Diseases in New York.

Even these figures furnish but a poor basis for an estimate of the prevalence of vereneal diseases in New York. Only a third of the physicians have given statistics, and there is no certainty that the figures do not in some instances represent duplication of visits by the same patient. It is not safe to multiply the total reported cases by three, because many physicians do not treat venereal diseases and the specialists for the most part were very careful to respond fully to the inquiry. It is safe to assume, however, that in a city as large as New York in which no attempts have ever been made to limit the transmission of venereal diseases, and in which, up to the present time, their treatment by hospitals and dispensaries has been more or less perfunctory, the number of cases must be very large indeed and that this number must continually tend to increase. The terrible ravages of syphilis are best appreciated, perhaps, by those physicians outside of venereal clinics who are connected with ophthalmological and neurological institutions, where the later stages of syphilis, the cases of optic atrophy, locomotor ataxia and general paresis are constantly in evidence. In a certain general medical clinic the Wassermann reaction performed indiscriminately as a matter of routine in a series of cases was positive in 20 per cent. The statistics recently promulgated by the Department of Health show conclusively that while the death rate of persons under forty-five has greatly diminished during the past thirty years, the death rate of persons over forty-five has increased and that this increase has been due in large part to the greater prevalence of disases of the heart, kidney, and nervous system. The interesting speculation at once arises as to whether this greater prevalence may not be due to an increase of syphilis which, as has been recently shown, is an active cause of diseases of the heart and kidneys, and, as is well known, is a prominent factor in nervous diseases. One of the foreign delegates to the Congress of Hygiene and Demography held in Washington in September, 1912, who afterwards visited many of the hospitals throughout the country is said to have stated that no where in the clinics of Europe had he seen anything like the large number of cases of syphilis and its final results as were found in the clinics of America, and that unless the medical authorities of the United States speedily took some efforts to combat its spread, syphilis would cause the deterioration of the American people. This statement of a mild and friendly critic coupled with such statistics, as we have, meagre though they are, certainly furnishes food for profound reflection.

### The Opposition and Its Rebuttal.

Nearly all the arguments which have been advanced by those opposed to the policy of public registration and sanitary surveillance are identical with those unsuccessfully advanced against registration of tuberculosis, when that procedure was inaugurated by the Board of Health in 1893, and it may safely be stated that very few of the physicians, who at that time advanced such arguments against the registration of tuberculosis, now entertain the same opinion. Some of the points raised in opposition may be briefly mentioned.

First it is urged that venereal disease, though often unjustly, is regarded as a reproach and a stigma. It may injure an individual's reputation and interfere seriously with his or her means of livelihood. Any knowledge obtained of it through professional sources is pre-eminently confidential, and should not be placed on public, or semi-public, or even private record except for purposes of treatment by those immediately concerned ,with the care of the case and bound by professional and legal obligations to absolute secrecy. It is a confessional matter as sacred as admissions made to a priest; and the rights involved and the duties entailed are as binding in the case of an unfortunate pauper as in that of the richest citizen of the land.

In this connection, while the general thought is doubtless sound, it must not be forgotten that the regulation of the Board of Health provides for absolutely no more publicity of records than exists at the present time in every hospital. The argument would seem to imply that the occurrence of venereal infction among hospital patients is kept absolutely secret. This, however, is not the case excepting in private patients, for the medical history, as well as the diagnosis, is almost invariably accessible to the entire hospital staff, including not only the physicians and nurses in charge of the case but also certain lay employees engaged in clerical work in the office. The Board of Health recognizes that information concerning the occurrence of venereal disease is preeminently confidential and has placed such restrictions about the keeping of its records of these cases that the information is safeguarded to a far greater extent than is the case in the hospitals.

This same fact of the careful restrictions surrounding the records should also be sufficient answer to the fears of those who are justly concerned to protect the "innocent" victim. It is urged that a large proportion of the diseases due to venereal infection, estimated at from 10 to 30 per cent., occur in perfectly innocent people, as in the wife who has gonorrhoeal disease of the pelvis acquired through infection by her husband, or in the infant who is born with hereditary syphilis. If no distinction is made between these and ordinary acute cases, it is said that an individual who is doubly unfortunate may have a permanent and essentially unjust record made against him or her. But, as a matter of fact, since the public records are practically inaccessible, even within the department to all except a single official in charge of the work, it is not justifiable to speak of a permanent record being made against a patient.

The opponents, however, are afraid that even if the secrecy of the reports can be successfully maintained under the present laws as to publicity of records, it can not be guaranteed against the uncertain future which may bring about new regulations, or new court interpretations of the old ones, or a change in the personnel of the health authorities which may at any time rend the veil of secrecy. The "dossier," they say, is there, officially and permanently. It may be lost or stolen and used for blackmailing, and thus be the cause of suffering and misfortune.

In reply, it may be pointed out that in the future, as at present, this work in the Department of Health will be in the hands of officials who are all medical men and well versed in medical ethics. Not only are the names and addresses of the patients kept confidential at the present time, but according to a recent interpretation of the highest courts (Allen v. Dept. of

Health, City of New York, affirmed by Court of Appeals, March, 1912, 205 N. Y. Reports, page 158) there is no way in which publication of information of this kind could be forced. So far as the records being lost or stolen is concerned, the same might be said about many other records of the department, for example, the records of illegitimate births. The fact, however, remains that the department records have always been efficiently safeguarded.

It is also said that the mere compilation of statistics in venereal diseases has little or no value. It has been carried out for years in various parts of the world without adding to our means of coping with these affections and without diminishing their frequency. Even if such compilation is desirable, a revelation of the identity of each sufferer is not required. It is an elementary truth that there can be no value in a mere compilation of statistics. The department has not undertaken this work in order to keep clerks or adding machines busy. The notification and registration of communicable diseases always has as its object supervision of the cases, and, while it is impossible to forecast definitely the various phases in which this supervision will be exercised, it will suffice to call attention to the enormous work that has been done in controlling tuberculosis along administrative lines.

Finally, it is urged that the ultimate effect of notification as now proposed will be to relegate not only venereal practice in the more restricted sense of the term, but all the multitude of remoter affections dependent on these types of disease, to the less reputable and less law abiding members of the profession, or to those outside its bounds. It will also tend to send those so afflicted to localities where the confidential relationship of physician and patient can be observed, or, finally, to promote the dangerous practice of self medication. Syphilitics and gonorrhoics of the poorer classes will avoid our public institutions in favor of individuals or "institutes" that will not report them, or will do so under fictitious names and addresses. Our dead walls will be plastered again with quack advertisements for the drug store will report no cases and even the Board of Health can hardly make a patient report himself. The ultimate gainers from the measure will be the quacks and patent medicine venders and the ultimate losers will be the unfortunate sufferers themselves.

To hold that this truly unfortunae state of aqairs would result from the enforcement of notification is to state a matter of pure opinion unsupported by any evidence whatsoever. In fact, there is every reason to believe that with the medical adviser, who is to be stationed in the Department of Health diagnostic clinic, just the contrary effect will be produced and that a larger proportion of venereally affected patients than ever before will be turned over to competent medical authorities for treatment.

### Legal Liability of Physicians.

One more argument is perhaps worthy of special consideration. It is held that the allegation of venereal disease may be and is a ground for damage and divorce suits and a basis for blackmail and private revenge. Legal or illegal efforts in any of these ways may be directed against the reporter or those responsible for him as well as against the party or parties more directly concerned. Medical men in the public service should not be compelled to put on accessible and uncontrollable record apparent facts that may involve the life and happiness of all concerned, and may be terribly misused. A diagnosis is merely an individual opinion, and may be open to review.

In the first place, in answering this contention, it should be said that the possibility of such consequences does not, at the present time, deter the hospital authorities from entering in their case histories statements concerning the previous occurrence of a venereal infection and statements regarding the diagnosis of the disease, and just as little as these records are at the present time used for the purpose of damage and divorce suits, or made the basis for blackmail and private revenge, just so little would the Department of Health records be used for this purpose. In fact, because of restrictions placed about the venereal records in the Department of Health, there is no possibility of their being used for the purposes mentioned.

As to the liability of physicians reporting cases observed in their private practice, the law seems to be entirely clear. It is true that to charge a person with an infectious disease is actionable. To make the reports referred to, however, to public officers for the purpose of enabling them to check the spread of the disease is a privileged communication, especially when the ordinance or resolution requires such information to be furnished. There can be no recovery in actions for libel or slander without proof of malice and this underlies all actions to recover damages in the class of actions mentioned. Malice is sometimes presumed. A privileged communication is one made under such circumstances as to rebut the presumption of malice and where the communication is made in the performance of a duty imposed by law the presumption of malice is at once rebutted and no action to recover damages would lie. So that while ordinarily to charge a person with having a venereal disease would be actionable, as the law stands to-day proof of the truth of the charge is a complete defense. A physician in making a statement of the condition of his patient would at the most be guilty of a breach of professional etiquette for he would make no such statements concerning a patient as a rule, unless, as a matter of fact, the patient had the disease. Of course, the lips of a physician are sealed in a civil action or special proceeding and he may not disclose information acquired by him which was necessary to enable him to act, but the courts also hold that where a crime is involved and a physician makes an examination he may testify to the facts and it is generally understood that the prohibition referred to does not apply to criminal procedures causing the death of a patient. Where a woman was prosecuted for performing a criminal operation and upon the patient being taken to the hospital and examined, the physician making the examination was allowed to testify against the objection of her counsel.

An aspect of this situation which is particularly emphasized is that justification is always a bar to an action for libel or slander. By justification is meant the truth of the matter charged so that if a physician reports a case of venereal disease and he is charged with libeling the individual, upon the physician proving that the person had the venereal disease he makes a complete answer and one that entitles him to a verdict in his behalf. In other words, the truth of a libel, is a complete bar to a prosecution.

In a word, then, the Department of Health holds that a physician in making an official report of a case of venereal disease is not actuated by malice; that the report is a privileged communication; that the act of the physician in making such report does not render him liable to prosecution of any kind; that he is performing a public duty required by law and that the regulation requiring such reports to public officials is reasonable and designed to protect general public health; and furthermore, that a physician making such a report may plead the truth of the report as a complete bar to any prosecution.

THE FUTURE OF ADMINISTRATIVE CONTROL.

As already stated, the object of the Department in requiring the administration of venereal diseases is to lay the foundation for a system of actual supervision of cases. The development of the particular methods of such supervision is a matter for the future and the Department is not yet ready to lay down in detail the exact plan and method of this surveyance. As an interesting suggestion in this connection, which also illuminates the attitude of private physicians toward the work of the Department it may be pertinent to quote, without implying official acceptance or approval of the plan described, the following editorial which recently appeared in the New York Medical Journal:

> * * "Similar methods (home visitation in tuberculosis) might well be employed against venereal disease, than which there is no greater menace to the public health. And since, as already stated, the object of home visitation is to appeal directly to the patient concerned and overcome his difference and ignorance, it would seem advisable to confine this work, as in tuberculosis, to persons treated in free dispensaries and hospitals. Physicians, and not nurses, should be employed to make these visits, and particular emphasis be laid on instruction as to the necessity for proper and adequate medical treatment, and as to the spread of infection. It is clear that this affords a definite field of activity for the public health authorities, and one constituting a useful and necessary adjunct to the work of private physicians, hospitals and dispensaries. The medical profession has strongly objected to the health authorities undertaking the TREATMENT of the venereal diseases by means of dispensaries, but the plan here outlined admirably supplements, and in no way conflicts with the work of existing agencies. It is evident, from what has been said, that the only effective means of reaching a very large part of the population will be closed to the public health administrator if home visitation is interfered with, and it is equally clear that there can be no home visitation without notification to and registration of the cases by the health authorities. It may be that the name "clinic" or "dispensary," which is proposed by the health department for its diagnostic stations, is objectionable to the practicing physician, but it is clear that the department has no intention of interfering with the practice of the physician; on the contrary, patients will be advised, if the case has been diagnosticated as syphilis, to consult a physician and to follow his advice, and as the visiting physicians employed by the health department are not permitted to solicit such patients, these patients will go back to their own physicians."

### Diagnostic Clinics.

The Department of Health of New York City has for some months conducted two diagnostic clinics for venereal diseases, where physicians may send patients for Wassermann tests for syphilis, for the complement deviation test for gonococcus infection, and for microscopical examination for treponema pallida. Under no circumstances are the results of these examinations communicated to anyone but the physician sending the cases. All the examinations are made free of charge. That the diagnostic aid thus afforded practicing physicians is appreciated, is evident from the rapid increase in the number of cases examined since the inauguration of this work.

In order to extend the usefullness of these "clinics" in combating the spread of the venereal diseases, a specially trained physician has been appointed to act as "medical adviser" to the patients attending the "clinic." Under no circumstances will he treat the cases. His duties will consist in advising patients as to the necessity of proper medical care, the dangers of self-treatment, and the importance of obeying the directions of their physicians. Upon request, the "medical adviser" will also instruct the patient regarding the nature of venereal infections, their mode of spread, ordinary course, complications and far-reaching effects, and in various questions of sexual hygiene.

To reach certain patients believed by the Department of Health to be inaccessible through other channels and to combat the quack treatment of venereal diseases, the following advertisement is to be placed in certain daily newspapers, and a sign similarly worded is to be placed in the toilets of elevated and subway stations and saloons.

> "Free advice regarding venereal diseases may be obtained at the Department of Health, 149 Center Street, Room 802, on Mondays, Wednesdays and Fridays, from 2 to 4 P. M., and on Tuesdays, Thursdays and Saturdays, from 9 to 11 A. M. All consultations strictly confidential."

The establishment of these venereal "clincs" has been disapproved of by some, on the ground that ample facilities for the free treatment of venereal diseases now exist. It is apparent that this objection is based on a complete misunderstanding of the function of the "medical adviser." From what has just been said it will be clear that not only will the medical advice given in no way interfere with the work of private physicians and dispensaries, but that it will, on the contrary, materially aid them in successfully treating their patients.

---

Mistakes in didagnosis are sometimes made by accepting a negative Wassermann reaction as exclusive of syphilis.

Pyelography will sometimes demonstrate the cause of a renal colic when radiography, cystoscopy and examination of the separated urines have shown nothing.

